# Effect of Propofol on Acid Reflux Measured with the Bravo pH Monitoring System

**DOI:** 10.1155/2013/605931

**Published:** 2013-04-22

**Authors:** Anupama Chawla, Eugenia Girda, Grace Walker, Frances Turcotte Benedict, Mila Tempel, Jeffrey Morganstern

**Affiliations:** ^1^Division of Pediatric Gastroenterology, Stony Brook Long Island Children's Hospital, Health Science Center T11-080, Stony Brook, NY 11794, USA; ^2^Department of Obstetrics and Gynecology and Women's Health, Belfer Educational Center, 1300 Morris Park Avenue, Room 501, Bronx, NY 10461, USA; ^3^Division of Gastroenterology, Stony Brook University Medical Center, Stony Brook, NY 11794, USA; ^4^Department of Pediatric Emergency Medicine, Alpert School of Medicine of Brown University, 2nd Floor, 55 Claverick Street, Providence, RI 02903, USA

## Abstract

*Background/Aim*. The aim of this study was to determine the effect of propofol on acid reflux as measured with the Bravo pH monitoring system. *Methods*. 48-hour pH tracings of 88 children were retrospectively evaluated after placement of the Bravo capsule under propofol. Comparisons between day 1 and day 2, as well as 6-hour corresponding segments from day 1 and day 2, were made. *Results*. The number of reflux episodes was significantly increased during the first six-hour period on day one as compared to day 2 (*P* = 0.006). The fraction of time the pH was <4 was also increased during this period, though it did not reach statistical significance. When comparing full 24-hour periods, there was no difference noted in either the number of reflux episodes or the fraction of time pH < 4 between day one and day two. *Conclusion*. Our data suggest an increase in gastroesophageal reflux during the postanesthesia period. This could be a direct effect of propofol, or related to other factors. Regardless of the cause, monitoring of pH for the first 6 hours following propofol administration may not be reliable when assessing these patients. Monitoring pH over a prolonged 48-hour time period can overcome this obstacle.

## 1. Introduction

Gastroesophageal reflux is characterized by the retrograde transit of stomach contents, including gastric acid into the esophagus. Clinical symptoms associated with it range from simple heartburn to aspiration pneumonia to ALTES (acute life-threatening events) in infants. The primary method for documenting and characterizing gastroesophageal reflux is a 24–48-hour pH monitor, either wired (simple pH probe) or wireless (Bravo system; Medtronic, Inc., Shoreview, MN, USA). Ambulatory extended esophageal pH monitoring is regarded as the standard test for establishing the presence of abnormal gastroesophageal reflux in children. Standard pH probe monitoring is performed by placing an esophageal catheter through the nose and advancing it to a precalculated distance above the lower esophageal sphincter (LES). Due to the morbidity and intolerability associated with the nasally placed pH catheter, the Bravo pH system has become a more commonly used procedure with comparable results and better tolerance among children.

The Bravo pH system consists of a small capsule which contains an antimony pH electrode, a radiotransmitter, and an internal battery. The capsule is attached to the mucosal wall of the distal esophagus and monitors pH, while transmitting data to a pager-sized device that may be easily attached to the patient's clothes. The disposable capsule usually falls off the esophageal wall in about 2 to 5 days and then passes through the gastrointestinal tract in another 2 days. The battery life of the capsule is about 7 days. The Bravo pH monitoring system monitors esophageal pH over a 48-hour period, which is the storage capacity of the device. The radio frequency (RF) is 433 megahertz, which has been approved by the Federal Drug Administration (FDA) for use in humans. There are no known long-term effects due to the brief presence of this RF device in the gastrointestinal tract. However, upper endoscopy and Bravo placement carry the risks of anesthesia, infection, bleeding, perforation, and chest pain. Compared to standard pH catheter devices, the overall safety of the Bravo probe, along with its high sensitivity (100%) and specificity (83.3%), makes it a reliable and well-tolerated assessment method for children ranging in age from 4 to 16 years [1].

At our institution, all wireless and some wired probes are placed under anesthesia. Intravenous propofol is our preferred method of anesthesia for the Bravo pH measuring device placement. However, it is unclear whether such sedation or the presedation fasting required may influence the frequency or magnitude of reflux during the study. 

Immediate effects of sedatives on gastroesophageal reflux, esophageal motility, and lower esophageal sphincter (LES) pressure have been previously studied. Galatos et al. in an animal study showed that gastroesophageal reflux, as evidenced by a decreased esophageal pH to less than 4, occurred in 16% of subjects who were anesthetized with thiopentone and propofol. In their study, reflux usually occurred shortly after the induction of anesthesia and had a mean duration of about 23 minutes [2]. However, Thorn et al. evaluated the effect of propofol on esophageal motility and LES pressure in healthy human volunteers. There was no statistical difference shown in LES pressure of subjects before and after propofol administration [3]. Other studies looking at the effect of conscious sedation on LES pressures have yielded mixed results [4–7]. In one study conducted on 19 infants and children, the effect of midazolam on LES pressure and motility was evaluated. No change in LES pressure or motility patterns was observed before and after midazolam administration [8]. 

Placement of Bravo measuring devices under conscious sedation or anesthesia has become the standard of practice in most US pediatric institutions. However, to the best of our knowledge, the effect of propofol, a commonly used anesthetic agent, on the results of the Bravo study has not been examined.

As we rely on pH monitoring for diagnosis and treatment of gastroesophageal reflux, it would be important to be aware of the possibility of false positive or negative readings secondary to recent sedation. Our results may help identify a postanesthesia period during which pH studies may not be reliable.

## 2. Methods

After obtaining approval from our institution's Committee on Research Involving Human Subjects, a retrospective analysis of the pH tracings of all children who successfully completed 48-hours of Bravo pH recording over the three-year period from 2007 to 2010 was performed. These included 88 subjects (46 males and 42 females) whose average age was 12.1 years with ages ranging from 4 to 18 years. All studies were completed at Stony Brook University Medical Center and conducted by the Division of Pediatric Gastroenterology. All subjects had IV propofol for the procedure as per protocol. 

All subjects had undergone esophagogastroduodenoscopy and Bravo esophageal pH testing for symptoms suggestive of gastroesophageal reflux. Indications for endoscopy and pH monitoring included persistent epigastric or substernal pain, persistent vomiting, belching, heartburn, chronic nocturnal cough or wheezing, and persistent throat clearance suspected to be caused by gastroesophageal reflux. 

### 2.1. Standard Bravo Probe Placement Protocol

The Bravo capsule is attached to the esophageal mucosal wall as per Bravo placement protocol. After identifying the *z*-line at the esophagogastric junction during endoscopy and noting its distance from the incisors on the endoscope, the endoscope is withdrawn. The prepackaged delivery system and capsule are then introduced into the esophagus to position the pH electrode at the distal end of the capsule at 87% of the endoscopically predetermined distance from the incisors to the *z*-line. The endoscope is reintroduced to visually verify capsule attachment.

### 2.2. Data Collection

The data from all 88 children who had completed 48-hour pH probe tracings measured using a Bravo capsule were analyzed. In our analysis, the data from the first 24 hours was compared to the data from the second 24-hour period. Each 24-hour recording was further subdivided into 6-hour segments for comparison. Six-hour segments from day 1 were compared to corresponding 6-hour segments on day 2.

## 3. Results

**Figure 1:**
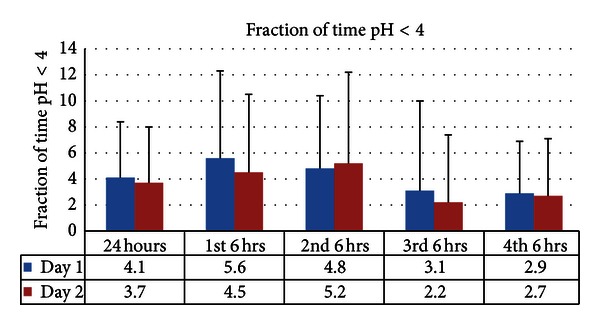
Increased fraction of time with pH < 4 during the first 6-hour segments of day 1 compared to the corresponding 6-hour segments of day 2 which did not reach statistical significance (*P* = 0.07). No difference observed when comparing 24-hour periods.

There were no statistically significant differences between the fraction of time the pH was <4 between the 24-hour period of day 1 and 24-hour period of day 2. Although not statistically significant, when data from corresponding segments were compared from day 1 to day 2, the first 6-hour segments of day 1 had increased fraction of time with pH < 4 compared to the corresponding 6-hour segments of day 2 (*P* = 0.07) (Figure 1). 

**Figure 2:**
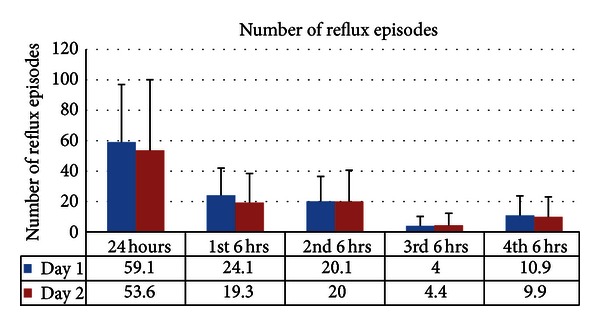
Significantly increased number of reflux episodes in the first 6-hour segments of day 1 compared to the corresponding segments of day 2 (*P* = 0.006). No difference observed when comparing 24-hour periods.

There was no significant difference in the number of reflux episodes between the 24-hour period of day 1 and 24-hour period of day 2. When comparing 6-hour segments from day 1 to the corresponding 6-hour segments from day 2, there was increased number of reflux episodes in the first 6-hour segments of day 1 compared to the corresponding segments of day 2 (*P* = 0.006) (Figure 2). 

## 4. Discussion

To our knowledge, this is the first study analyzing the effect of propofol on acid reflux as measured with the Bravo pH monitoring device. Anesthetic agents, including midazolam, diazepam, thiopentone, remifentanil, and propofol, have shown to have different effects on the LES pressure and esophageal motility [2–9]. Galatos et al. compared the effect of thiopentone and propofol on gastric esophageal reflux in cats [2]. pH was monitored throughout the length of the surgical procedure, approximately 30–60 minutes. The results demonstrated increased reflux with propofol but no significant change with thiopentone. Thorn et al. compared the effect of propofol with remifentanil and concluded that both anesthetics did not have a significant effect on LES pressure or esophageal motility [3]. Studies by Marsh et al. and Fung et al. evaluated the effect of midazolam on esophageal motility [4, 8]. While Marsh et al. reported an increase in LES pressure and decreased percentage of relaxations in patients with midazolam, Fung et al. concluded that midazolam did not change the LES pressure. These multiple studies of different anesthetic agents on reflux-related factors yielded variable and sometimes contradictory results. 

Our retrospective data analyzing pH tracings of 88 patients showed no difference in the fraction of time pH was <4 on day 1 compared to day 2. When this data was further analyzed comparing 6-hour segments on day 1 to the corresponding 6-hour segments on day 2, the 6-hour period on day 1, immediately following propofol, showed an increase in the fraction of time the pH was <4 compared to the corresponding 6-hour period on day 2 (*P* = 0.07). Although not statistically significant, we hypothesize that a larger sample size would result in statistical significance.

Regarding the number reflux episodes in the 24-hour period on day 1 compared to day 2, there was a slight increase but the values did not reach statistical significance. There was, however, a significant increase in the number of reflux episodes in the first 6-hour segments on day 1 compared to the corresponding segments on day 2 (*P* = 0.006). Therefore, most likely events related to anesthesia contributed to the increase in reflux episodes on day 1. 

Based on previous studies, it is unclear whether propofol has a direct effect on the esophageal motility and LES pressure. A recent study by Turan et al. concluded that propofol may cause some decrease in LES pressure at high concentrations of the drug administration [9]. None of the studies took into account possible secondary mechanisms involving factors such as level of activity, patient's position, and dietary intake. These variables were not controlled for in our study. It is possible that a prospective study with a larger patient population may help isolate the effect of propofol while controlling for these variables.

The increase in the number of reflux episodes noted during the first 6-hour period following placement of Bravo suggests that propofol or other related factors such as NPO status, endoscopy with air insufflation, decreased oral intake, and lack of activity after anesthesia administration could contribute to increased duration of acid reflux in the immediate postanesthesia period. Based on these results, pH monitoring in the first few hours after the placement of the Bravo capsule with propofol may not be reliable. However, prolonged monitoring, particularly over a 48-hour period, and perhaps disregarding the initial few hours, should overcome this obstacle resulting in an overall reliable study.
